# Communicating causality

**DOI:** 10.1007/s10654-015-0086-6

**Published:** 2015-10-07

**Authors:** Sonja A. Swanson

**Affiliations:** Department of Epidemiology, Erasmus Medical Center, P.O. Box 2040, CA 3000, Rotterdam, The Netherlands; Department of Epidemiology, Harvard T. H. Chan School of Public Health, 677 Huntington Avenue, Boston, MA 02115 USA

We epidemiologists have long recognized the importance of using rigorous causal inference approaches to design and analyze our studies. Causal diagrams comprise one such tool for formalizing assumed data-generating processes. And indeed, the ubiquity and importance of causal diagrams within epidemiology is evidenced by four articles presented in this issue of the *European Journal of Epidemiology* [1–4]. As epidemiologic studies are often used to inform clinical and policy decision-making, we have also understood the need to unambiguously communicate our studies’ findings amongst ourselves and across the disciplines with whom we collaborate. While others have taught and espoused how causal diagrams can guide and improve our study designs and analyses [5–7], perhaps one of the most transformative aspects of the current “revolution”—in the words of Porta et al. [4]—is that we have adopted tools that enhance the clarity of our study conclusions and the premises on which they rest.

## Causal diagrams as (formal) story-telling

When and why are causal diagrams useful? One of the most evident successes of causal diagrams is in supplementing story-telling. With a few arrows and letters, an investigator can tell a story of a data-generating process. For a reader fluent in causal diagrams, even a dauntingly complex story can now be quickly and fully digested. In this way, we have seen a series of “paradoxes” demystified, including proposed explanations for the so-called Berkson’s [8], birth-weight [9], obesity [10], and Simpson’s [11] paradoxes. Similarly, causal diagrams focused our attention on the structures of oft overlooked potential biases, such as biases due to time-dependent confounding in stratification-based analyses [12], mediator-outcome confounding in mediation analyses [13], selecting on treatment in instrumental variable analyses [14], and naïve per-protocol restrictions in randomized trial analyses [15]. Readers familiar with causal diagrams will recognize that many of these examples can be described as collider-stratification biases, and that, while some encompass previously recognized threats to validity, these potential biases were infrequently mentioned until their associated causal diagrams were drawn.

Beyond demystifying perplexing patterns or illuminating subtle problems that exist across many studies, causal diagrams can also facilitate debates regarding a specific study’s conclusions. Consider two investigators who are in disagreement over whether a specific study’s analysis and conclusions were appropriate. If these two investigators “speak DAG” (directed acyclic graph) then they may seamlessly convey their assumptions and ideas to one another with little fear of miscommunication. Perhaps the two investigators will realize they had different causal diagrams in mind, and that favoring one analytic approach over another depends on which causal diagram is drawn—and thus on particular assumptions that, undrawn, might have suggested favoring a different analysis. Perhaps they will even be able to collect further data to help settle on which causal diagram—which set of assumptions—is more reasonable. Such discussions, which can be cumbersome and confusing without a formal language, can take place quickly and explicitly when supplemented with causal diagrams.

In these ways, a causal diagram, like a picture, is worth one thousand words. Unlike artwork, however, where the “thousand words” convey a subjective perspective, a causal diagram should convey exactly the thousand words its creator and all other fluent readers would attribute to it. Causal diagrams are useful because they facilitate precise communication, but ignoring the formal rules that govern them can lead to miscommunication. For some examples of this, we can turn to an article in this issue of the *European Journal of Epidemiology* in which Greenland and Mansournia [3] caution how failing to read a causal DAG as encoding only structural (not random) confounding or failing to be explicit about faithfulness when presumed can lead readers of a causal diagram to perceive a different “thousand words” than intended.

As with any tool that can streamline communication, there is also a danger of causal diagrams providing a false sense of security when they are constructed without investigators applying deep thought and subject matter knowledge. To see this, consider the use of causal diagrams in the context of instrumental variable analyses. Many epidemiology studies with instrumental variable analyses redraw the same textbook instrumental variable causal diagram to justify their analysis, yet the story is rarely as straightforward as the one depicted in that causal diagram. Hernán and Robins [16], Swanson et al. [14] and VanderWeele et al. [17] have presented expanded versions of this standard graph that illustrate relatively subtle yet potentially common ways in which bias could arise. Thus, redrawing the textbook version of a causal diagram may oversimplify the likely data-generating process and even offer false comfort when applied to a specific study. Of note, some have argued that causal diagrams are not useful in the context of instrumental variable analyses because “the” DAG seems so simple that drawing it does not add to our understanding of the process [18]. While causal diagrams (arguably) add less to our understanding of what is a true instrument, we have seen many examples of causal diagrams adding substantially to our understanding of what is not an instrument.

If two epidemiologists “speak DAG” fluently and think deeply while constructing causal diagrams, they can cleanly convey their premises and ideas to one another with little fear of miscommunication. However, many of us are not fluent in causal diagrams. Moreover, fluency or even familiarity with causal diagrams is currently rare among the broad range of medical researchers, clinicians, and policy-makers with whom we work. While our field would doubtlessly benefit from having more fluent speakers, we as a field ought to ask ourselves: should fluency in causal diagrams be a requisite in our training and communication standards?

## The case for causal inference “multilingualism”

Causal diagrams are attractive because they facilitate clear communication. Of course, the same argument can be made for other formal representations, including the counterfactual outcome framework that DAGs are linked with in this issue [2]. Should epidemiologists favor one framework over another? Ultimately, translations between these representations are achievable, as evident by the mathematical equivalencies between the DAG-based do-calculus and the counterfactual-based g-formula [2, 19–21]. Nonetheless, in our day-to-day work as epidemiologists, an argument could be made that learning to both “speak DAG” and “speak counterfactuals” will deepen our own comprehension of our subject matter.

Being well-versed in multiple formal representations of causality can lead to not just clearer but also more efficient communication. For example, some assumptions (e.g., directionality or monotonicity of treatment effects) are readily stated via counterfactuals but require augmentations to our causal diagrams. Indeed, defining causality without mention of counterfactual outcomes—as counterfactuals are not immediately apparent in DAGs, although they do take center-stage in single-world intervention graphs [22]—may seem at times like learning a language with one less tense. On the other hand, particularly in high-dimensional data, translating a data-generating process from a causal diagram to a list of independencies expressed with counterfactuals can be onerous—why do we need to use so many phrases to express something that would otherwise be succinctly (and appropriately) stated in a diagram? Each representation has advantages, and being facile with multiple formal representations allows us to capitalize on the benefits of all.

Considering the benefits of causal inference “multilingualism” lends itself to another question we should ponder: should every epidemiologist learn every language? As a corollary question, what would be the benefits of a causal inference Esperanto that explicitly combines the best of graphical and counterfactual language? Perhaps the future of succinct and clear communication in epidemiology lies in single-world intervention graphs [22].

## Conclusion

Regardless of the framework in which it is couched, inferring causality comes down to combining data and assumptions. As epidemiologists, we make causal inferences all the time. Consequently, it is our responsibility to communicate effectively the assumptions we are making and the way in which we combine assumptions with data. Science benefits when communication is flawless—i.e., when our premises are precisely and transparently stated, and our results are accurately interpreted. In embracing causal diagrams, we are indicating our commitment to unambiguous communication.
